# In Vitro Comparative Evaluation of Newly Produced Desensitizer, Chlorhexidine and Gluma on Bond Strength and Bond Longevity of Composite to Dentin

**DOI:** 10.30476/DENTJODS.2019.77756.0

**Published:** 2020-06

**Authors:** Reza Davalloo, Seyedeh Maryam Tavangar, Heshmatollah Ebrahimi, Farideh Darabi, Shima Mahmoudi

**Affiliations:** 1 Dental Sciences Research Center, Dept. of Restorative Dentistry, Faculty of Dentistry, Guilan University of Medical Sciences, Rasht, Iran; 2 Dept. of Medicinal Chemistry, School of Pharmacy, Guilan University of Medical Sciences, Rasht, Iran; 3 Postgraduate Student of Restorative Dentistry, Faculty of Dentistry, Guilan University of Medical Sciences, Rasht, Iran

**Keywords:** Bond Strength, Chlorhexidine, Gluma

## Abstract

**Statement of the Problem:**

Etching process on dentin can activate matrix metalloproteinase (MMP) which hydrolyze organic matrix of demineralized dentin. Gluma and chlorhexidine could inhibit the activation of MMP.

**Purpose:**

The aim of the study was to evaluate the effect of a new desensitizing material consisting of Gluma and chlorhexidine together on the shear bond strength and bond durability of composite restorations.

**Materials and Method:**

One hundred and twenty caries-free extracted premolars were sectioned horizontally from one third of the coronal crown to expose flat dentin surface and randomly divided into 4 groups. In the control group, no surface treatment was used. In the first group chlorhexidine (CHX) 2%, in the second group, new material (NM) and in third group Gluma desensitizer (GD) was applied after etching and before bonding(total-etch bonding system). After the bonding process, the composite was placed on the surface of the samples using a cylindrical mold. Then, the shear bond strengths of half of the specimens were measured after 24 hours and the other half after 6 months of storage in distilled water and thermocycling. The failure types of specimens were evaluated with a stereomicroscope. Data were analyzed using One-way Anova and Tukey's Post Hoc tests in SPSS software.

**Results:**

After 6 months, the bond strength decreased in all groups and differences were statistically significant (*p*= 0.002).The highest shear bond strength was observed after 6 months in the NM group and the GD group with no statistically significant difference. The 24-hour bond strengths were not significant between groups. Mix failure had the highest rate in all groups.

**Conclusion:**

It can be concluded that the effect of combination of chlorhexidine and Gluma on maintaining the integrity and strength of bond over time is similar to Gluma compound alone and they have better effect than chlorhexidine.

## Introduction

Nowadays, because of the increased demand for esthetics from patients, the use of resin composites has increased for posterior teeth restoration [ 1 ]. For success of composite restorations, creating a durable bond between composite resin and dental structure is essential [ 2 ]. Although the strength of the bond to the enamel is stable over time, this is not true for dentin, which has reported to be decreased 30-40% after 6 months and 60-70% after 1 year [ 3 ]. 

In most studies, bond integrity has been investigated in short periods, such as after 24 hours. Several studies have investigated the durability of resin and dentin bond after long-term storage in water, which resulted in a reduction in bond strength after 6 months immersion in water [ 4 ]. The researchers believe that the reduction of bond strength over time is due to the hydrolysis of adhesive resins and the activity of collagenases [ 5 ]. 

Endogenic matrix metalloproteinases (MMPs) are activated after the use of the acid etchant, followed by the hydrolysis of the hybrid layer collagen, which cause decrease in bonding durability [ 6 ]. Therefore, the use of a substance that has the ability to inhibit MMPs is valuable to maintain bond strength over time [ 7 ]. Materials such as chlorhexidine, Gluma, EDTA, tetracycline and quaternary ammonium salts, such as 12-methacryloyloxydodecyl pyridinium bromide, have this ability [ 7 - 9 ]. 

The effect of chlorhexidine 2% on dentin bond strength and reduction in the degradation of hybrid layer over time (6 months of aging) was reported [ 10 ]. Unfortunately, chlorhexidine is water-soluble [ 11 ] and is not copolymerized with resin .It is washed away from the inside of the hybrid layer during one to two years, resulting in collagen degradation. It has been reported that the protective effect of chlorhexidine on the bond remains for up to nine months, and after 18 months, this effect has not been seen [ 3 ]. 

Gluma adhesives are the first bonding agents containing organic compounds of aldehyde. Gluma contains 5% glutaraldehyde and 35% HEMA (hydroxyethylmethacrylate) and has antibacterial properties .It is used as a substance to reduce post-operative sensitivity [ 3 , 7 ]. Applying Gluma (Gluma desensitizer liquid) for 60 seconds on etched dentin surface has been reported to inhibit 86% of endogenous MMPs(by cross-linking of MMPs molecules), which increases the stability of the resin-dentin bond *in vivo* [ 3 ]. 

To benefit from the desired properties of chlorhexidine and Gluma simultaneously, in this study a new compound containing chlorhexidine and Gluma in ethanol solvent has been investigated. The purpose of this study was to evaluate the effect of this new material on the shear bond strength and bond durability of composite restorations.

## Materials and Method

In this experimental study, 120 extracted non-carious human premolar teeth (due to orthodontic treatment) were collected. They were cleaned and disinfected with 0.5% chloramine-T solution (Fisher chemical; Fair lawn, NJ, USA) and then stored in distilled water at 4° C in accordance with ISO 11405. The one-third of coronal crown of all teeth were sectioned horizontally (perpendicular to the long axis of the tooth) using water-cooled low-speed saw (Isomet, Buehler Ltd, Evanston, IL, USA), to expose dentin. Then, by using a 600-grit silicon carbide abrasive paper (Snam Abrasives Pvt. Ltd., India) the surfaces were polished.

The teeth were then randomly divided into four groups, three experimental groups and one control group. Table 1 displays the data concerning the manufacturer as well as the composition of all materials used in this study. In the control group, no surface treatment was used. In all groups, acid etching (FGM, Brazil) was applied for 15 seconds then washed and blot dried. In all experimental groups, surface treatment materials were applied after etching and before bonding.

**Table 1:** Materials used in this study

Materials	Composition	Manufacturer
Ambar	Active ingredients: 10-Methacryloyloxydecyl dihydrogen phosphate, Methacrylic monomers, Co-initiators and Stabilizers. Inactive ingredients: inert load (Silica Nanoparticles) and vehicle(ethanol)	FGM (Brazil)
Opallis	Active ingredients: Bis-GMA monomers, Bis-EMA, TEGDMA, UDMA, co-initiator, and silane. Inactive ingredients: Silanized barium-aluminum silicate glass, pigments and silica	FGM (Brazil)
Gluma Desensitizer	(2-Hydroxyethyl)methacrylate, glutaraldehyde, purified water	GD, Heraeus Kulzer South bend (Indiana)
Chlorhexidine	2 % Chlorhexidine	PPH CERKAMED Wojciech Pawlowski (Poland)

In the first group (CHX group), chlorhexidine 2% (CHX, PPH CERKAMED Wojciech Pawlowski, Poland) was applied on etched dentin surface for 60 secon-ds with microbrush, the excess was removed gently. In the second group (NM group), new material (NM) containing 35% HEMA, 5% glutaraldehyde, and 2% chlorhexidine(all dissolved in ethanol) was applied on etched dentin surface for 60 seconds with microbrush and the excess was removed with blot dry technique. The method of production of this new material is described later in this section. 

In the third group (GD group), Gluma desensitizer (GD, Heraeus Kulzer, South bend, Indiana) was applied on etched dentin surface and after 30 seconds and the excess was removed gently with tissue paper, leaving the surface visibly wet. The bonding used for this study was Ambar (FGM, Brazil, total-etch system) and was applied according to manufacturer instruction.

**Figure 1:**
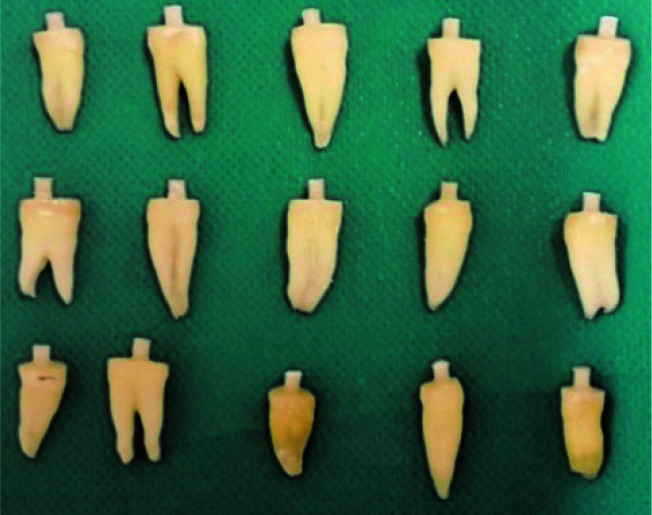
Sample preparation

The bonding was applied in all groups, then the bonded surfaces of the samples were polymerized with LED light-curing unit (Bluedent LED smart, Bulgaria) at 1200 mW/cm2 intensity, controlled by a radiometer (RD-7, Paulo, Brazil) according to manufacturer instruction. Then a cylindrical mold (height: 3mm & diameter: 3mm) was placed and fixed on the smooth dentin surface using sticky wax while the specimens were carefully isolated. Then the composite (Dentin A_2_ shade, FGM, Brazil) was placed incrementally (each increment was 1.5mm) in the mold using a suitable condenser, and each layer was cured for 40 seconds at 1200 mW/cm2 light intensity (Figure 1). Subsequently, half of the teeth in each group separated for immediate shear bond strength (SBS) measurement and were stor-ed in distilled water for 24 hours at room temperature. The SBS of these samples was measured in a universal testing machine (STM-20, SANTAM, Iran).

**Figure 2:**
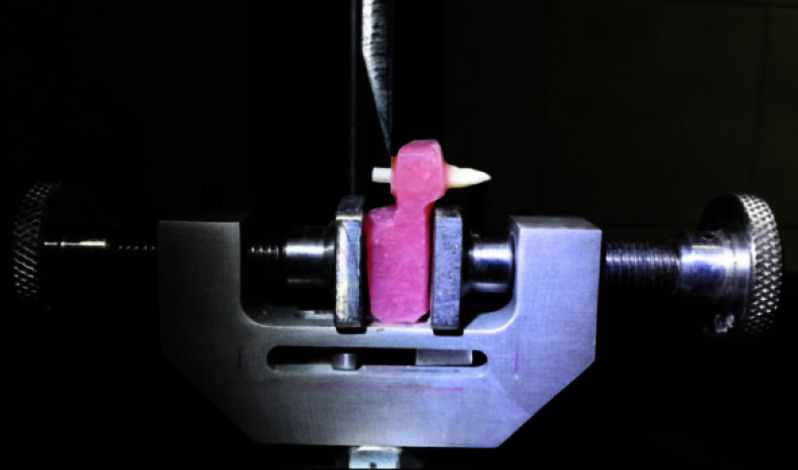
Evaluation of samples in terms of shear bond strength

The specimens were subjected to force at the tooth-composite interface, parallel to the bonded surface, utilizing a stainless steel rod with a sharp blade of 2.5 mm diameter at the speed of 0.5 mm/min until fracture occurred (Figure 2). The remaining specimens were stored in distilled water at room temperature for 6 months, then placed in a thermocycler machine and subjected to 5000 cycles (5°C and 55°C with 15 seconds of dwell time for each bath and 15 seconds of transfer time). It should be noted that based on previous studies, every 10,000 cycles are equivalent to one year of aging [ 12 ]. Then SBS of these specimens were measured with universal testing machine, as previously described.

The samples were examined under a stereomicroscope (Olympus DF Plapo 1X, Japan) at 20X magnification to evaluate the type of fracture. Types of fracture are define as (1) adhesive, when more than 90% of the bonded surface between the dentin and the composite resin was fractured; (2) cohesive, when more than 90% of the fracture occurred in either the dentin or the composite resin, and (3) mixed, when both adhesive and cohesive types have occurred.

For statistical analysis, all data were analyzed using SPSS software version 22 with descriptive statistics of mean and standard error. One-way ANOVA inferential test and Tukey's post-hoc test was performed for SBS. Chi-Square tests were used to analyze the failure types. A significant level of 0.05 was used for all tests. 

### New material production method

To prepare 25ml of a solution containing glutaraldehyde 5% and HEMA 35% and chlorhexidine 2%, the amount of 7.7ml of standard solution of HEMA with purity of 97% and 3.9ml of purified glutaraldehyde 25% with 2ml of 2% chlorhexidine were mixed. To investigate the chemical reaction of these materials, each solution was first separately detected in an ultraviolet–visible spectrophotometer (UV/VIS Spectrometer lambda 25, PerkinElmer, USA), and the wavelength-absorption diagram of each substance alone was recorded. Then the mixture of three substances was placed in the device and a mixture diagram of these substances was recorded. It should be noted that all stages of construction of new material were carried out by an experienced pharmacist.

## Results

**Figure 3:**
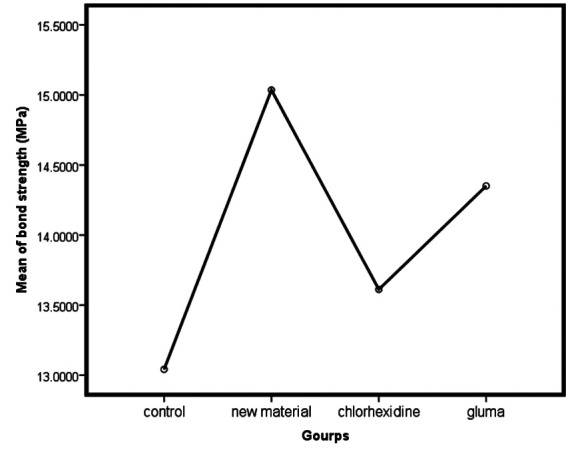
Mean shear bond strength of groups after 24 hours

**Figure 4:**
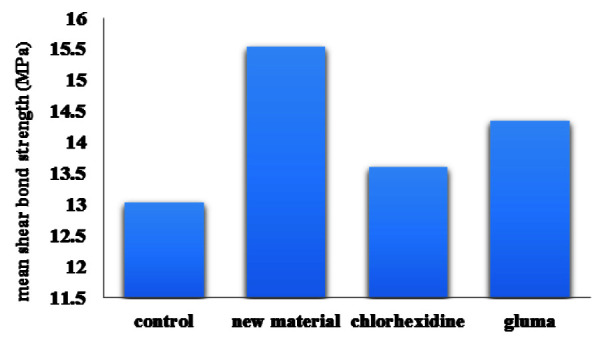
Mean shear bond strength of groups after 24 hours

**Figure 5:**
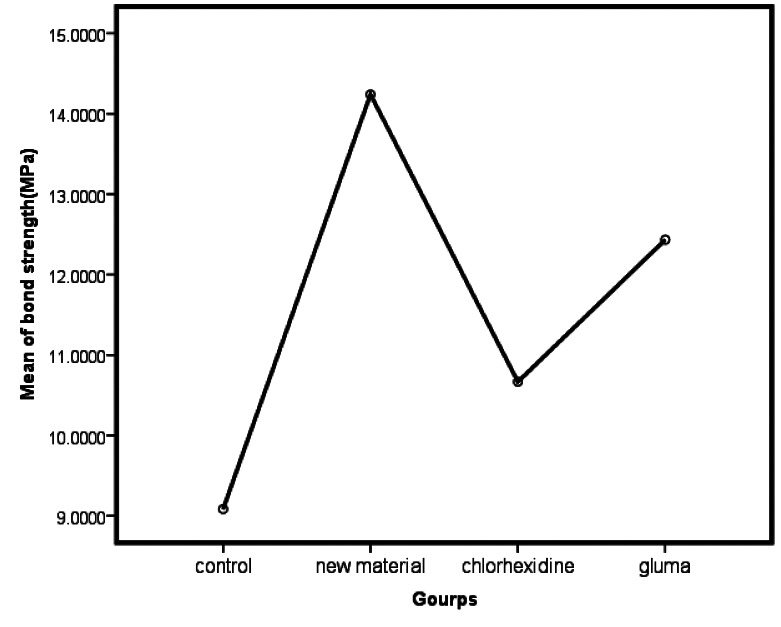
Mean shear bond strength of groups after 6 months

**Figure 6:**
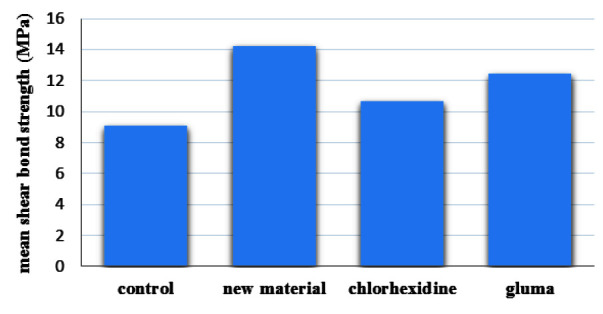
Mean shear bond strength of groups after 6 months

The mean SBS of composite to dentin in different groups after 24 hours is demonstrated in Table 2, Figures 3 to 4. Statistical analysis showed that SBS difference between groups was not significant (*p*> 0.05) and the highest SBS was observed in the NM group and the lowest in the control group. The mean SBS after 6 months has been demonstrated in Table 3, Figure 5, and Figure 6. The difference in SBS between groups was significant (*p*= 0.001). Multiple comparison between the groups using the Tukey post hoc test (Table 4) showed that SBS has a significant difference in the NM group with the control group (*p*= 0.001) and the CHX group (*p*= 0.01), but there was no significant difference with GD group (*p*= 0.19). 

**Table 2:** Mean shear bond strength(standard error ) of the study groups after 24 hours

Groups	Groups	Number of samples	Significant differences
Control	Control	15	*p*> 0.05
New Material	New Material	15	
CHX	CHX	15	
GD	GD	15	

**Table 3:** Mean shear bond strength(standard error ) of the study groups after 6 months

Groups	Bond strength, MPa (standard error)	Number of samples	Significant differences
Control	9.08(1.03)	15	*p*= 0.002
New material	14.24(0.64)	15	
Chlorhexidine	10.67(1.03)	15	
Gluma	12.43(0.52)	15	

**Table 4:** Multiple comparison of shear bond strength among groups after 6 months

(I) group	(J) groups	Mean Difference (I-J)	*p*
Control	New material	-5.1563600*	0.000
	Chlorhexidine	-1.5841000	0.240
	Gluma	-3.3497200*	0.015
New material	Control	5.1563600*	0.000
	Chlorhexidine	3.5722600*	0.010
	Gluma	1.8066400	0.181
Chlorhexidine	Control	1.5841000	0.240
	New material	-3.5722600*	0.010
	Gluma	-1.7656200	0.191
Gluma	Control	3.3497200*	0.015
	New material	-1.8066400	0.181
	Chlorhexidine	1.7656200	0.191

Tukey HSD analysis.

* the mean difference is significant at the 0.05 level

There was no significant difference between mean SBS in the control group with the CHX group (*p*= 0.445). However, there was a significant difference between the control group and the GD group (*p*= 0.015). In Table 5, the mean SBS of the samples was compared in term of time of storage.

**Table 5:** Comparison of the mean shear bond strength among groups based on time

Mean bond strength, standard error (MPa)	Number	Time	Group
15.55(0.84)^a^	15	After 24 hours	New material
14.24(0.64)^a^	15	After 6 month	
13.21(0.97)^a^	15	After 24 hours	Control
9/08(1.03)^b^	15	After 6 month	
13.61(0.84)^a^	15	After 24 hours	Chlorhexidine
10.67(1.03)^b^	15	After 6 month	
14.35(0.97)^a^	15	After 24 hours	Gluma
12.43(1.03)^a^	15	After 6 month	

As seen in the table, the difference in bond strength after 24 hours and 6 months was not significant in the NM and GD group, but was significant in the control group (*p*= 0.009) and the CHX group (*p*= 0.036). In all four groups, the mean bond strength was decrease after 6 months. The analysis of type of fracture was performed using Chi-Square Tests and is shown in Table 6. As can be seen, no cohesive fracture was observed in the samples. As far as the type of fracture was concerned, the most frequent type of fracture in all four groups was the mixed type. There was no significant difference in type of fracture among the samples (*p*= 0.237).

**Table 6:** Type of fracture analysis

Groups										Total
Control 1*			CHX 1	Gluma 1	New 1	Control 2	CHX 2	Gluma 2	New 2	
Fracture type	Adhesive	8(53.3 %)	8(53.3%)	8(53.3 %)	5(26.7%)	8(53.3%)	7(46.7%)	7(46.7%)	4(33.3%)	55
	Mixed	7(46.7 %)	7(46.7%)	7(46.7%)	10(73/3 %)	7(46.7%)	8(53.3 %)	8(53.3%)	11(66.7%)	65
Total		15(100 %)	15(100%)	15(100%)	15(100%)	15(100%)	15(100%)	15(100%)	15(100%)	120

* Control 1: control group after 24 hours, CHX 1: chlorhexidine group after 24 hours, Gluma 1: Gluma group after 24 hours, New 1: new material group after 24 hours. Control 2: control group after 6 month, CHX 2: chlorhexidine group after 6 month, Gluma 2: Gluma group after 6 month, New 2: new material group after 6 month

## Discussion

Degradation of hybrid layer of adhesion process to dentinal structure can occur over time because of endogenous proteases (MMP) attack. To prevent the effects of these MMPs on hybrid layer, various materials have been recommended, including chlorhexidine, an antimicrobial agent that can inhibit MMP 2, MMP 9, and MMP8. Chlorhexidine has also been reported to inhibit dentin cathepsins [ 3 ]. Another material that can inhibit protease activity is glutaraldehyde. Glutaraldehyde reacts with plasma proteins, such as albumin and causes them to precipitate on dentin surface. These sediments react with HEMA and form a mixture of Poly-HEMA copolymerized with glutaraldehyde-cross- linked-albumin. This deposition causes blockage of dentinal tubules; however, despite the formation of this sediment on the dentin surface, the penetration of adhesive monomers into dentin is accelerated by HEMA [ 13 - 15 ]. Glutaraldehyde has anti-microbial properties. It is also known as a cross-linking agent that can increase the un-cross-linked or mildly cross-linked collagen matrix resistance to enzymatic degradation. The mechanism of this action is to react between the aldehyde groups present in glutaraldehyde and the amine group in lysine and the hydroxy lysine remaining in the collagen. Glutaraldehyde, by improving the mechanical properties of dentin, minimizes bond degradation [ 13 ].

In this study, the effect of these two desensitizer agents together on dentin SBS and durability of bond was investigated. The results of this study showed that the use of chlorhexidine after dentin etching does not have any significant effect on SBS. Shafiei *et al*. [ 16 ] reported that the use of chlorhexidine prior to applying adhesive onto dentin significantly reduces the SBS. This difference was reported in Ercan *et al*. [ 17 ] as well as Meiers and Shook [ 18 ], which is inconsistent to the results of our study.

This could be due to the application of different adhesives used. The present study uses an etch-and-rinse adhesive. It has been reported that the type and composition of the adhesive system also affects the bond strength. Many studies have been done on the SBS of etch-and- rinse and self-etch adhesives. Some have reported that etch and rinse systems have a higher SBS [ 19 - 20 ] due to better resin hybridization and better infiltration into the collagen network [ 21 ]. 

It has also been reported that the use of CHX with self-etch adhesives reduces bond strength due to the limited penetration of adhesive into the dentin [ 1 ]. Zheng *et al*. [ 22 ] and Nishitani *et al*. [ 23 ] reported that the use of chlorhexidine with etch-and- rinse adhesives improved bond strength, but not in the self-etch group. Compos *et al*. [ 24 ] reported that using 2% chlorhexidine would reduce bond strength and concluded that it should not be used before self-etch adhesives. Breschi *et al*. [ 25 ] reported that the use of chlorhexidine even with low percentages (0.2%) on etched dentin could prevent collagen degradation by up to 12 months.

 It has been reported that CHX can inhibit MMPs and reduce the rate of degradation of the resin bond and dentin [ 26 - 28 ]. A study by Brackett *et al*. [ 10 ] reported that the decomposition of hybrid layer after 6 month in restorations using CHX after the etching process was slower than control restorations. However, CHX does not completely inhibit the degradation of hybrid layer over time [ 10 ], owing the loss of its properties over time [ 29 ]. 

Another problem affecting bond strength is the type of solution in which CHX is dissolved. Ali *et al*. [ 30 ] showed that the use of ethanol-based CHX has a negative effect on the bond strength of self-etch adhesives, but water-based CHX provides better bonding durability. The study of Ekambaram *et al*. [ 31 ] reported that using CHX 2% with ethanol-wet bonding would maintain the bond strength of the dentin after 12 months. Considering the newly made material in the present study, which contains CHX and Gluma in an ethanol solvent, the presence of ethanol might have a positive effect on bond strength. 

In the present study, the highest bond strength after aging was observed in the NM group, which was not significant from GD group. Maintaining bond strength after aging in both NM and GD group can be related to the presence of Gluma in their combination. In fact, the results indicate that the use of chlorhexidine and Gluma together in one composition, improves the stability of the bond strength over time.

As previously mentioned, the new substance contains 5% glutaraldehyde and 35% HEMA and 2% chlorhexidine dissolved in ethanol. The use of Gluma facilitates the expansion of the demineralized collagen network and increases surface energy, which facilitates the penetration of resin monomers into the demineralized dentin, thus improving bond strength [ 13 ]. 

Külünk *et al*. [ 32 ] reported that use of Gluma desensitizer (Heraeus Kulzer, Hanau, Germany) has positive effect on bond strength of resin cements.

Bedran‐Russo *et al*. [ 33 ] reported that using glutaraldehyde increases the modulus of elasticity and stiffness of the demineralized dentin, which increases over time and increases in higher concentrations of the substance. On the other hand, increasing the strength of the dentin matrix with crosslinking agents increases the strength and durability of the resin-dentin bond.

Ravikumar *et al*. [ 34 ] reported that the use of Gluma desensitizer after dentin etching could significantly improve the durability of bond in comparison with dry dentin, but was not statistically significant in comparison with wet dentin despite higher bond strengths. Soeno *et al*. [ 14 ] reported that the use of Gluma prior to the use of Panavia cement has no effect on bond strength. Sabatini *et al*. [ 13 ] also reported that the use of Gluma before using these adhesives did not significantly differ from the control group. However, a study by Huh *et al*. [ 35 ], reported that the use of Gluma prior to the application of ED primer and Panavia cement significantly reduced bond strength compared to the control group. The differences between the results of these studies can be due to the different adhesive used and different etching method, as well as differences in the method of measuring the bond strength.

Many studies have been done with scanning electron microscope (SEM) to determine the type of fracture in the specimens. In most studies, the fracture type was adhesive [ 35 - 36 ]. 

In this study, the stereomicroscope was used to evaluate the fracture mode and the most fracture type was mixed failure, although differences between groups were not statistically significant.

## Conclusion

It can be concluded that the effect of combination of chlorhexidine and Gluma on maintaining the integrity and strength of bond over time is similar to Gluma compound alone and they have better effect than chlorhexidine.
